# Bacillus of Tetanus

**Published:** 1890-02

**Authors:** 


Bacillus of Tetanus.—At the recent German Surgical Congress in Berlin, Dr. Kitasato, of Tokyo, Japan, demonstrated the tetanus-bacillus, and claimed that he had succeeded in obtaining this organism free from other bacteria, in experiments made in the Berlin Hygienic Institute. Tetanus serum was shown on agaragar, and after twenty-four hours the bristle-shaped tetanus-bacilli were present, along with various other micro-organisms. If the culture was now exposed to a water bath at 80° C. for half an hour, only the spores of the tetanus-bacilli retained their vitality; all the rest perished. Inoculations with the culture produced tetanus with the development of the typical bristle-shaped bacilli.—*London Medical Recorder*, Oct. 21, 1889.

Nurse.—" Oh, dear! I've given you a dose of ink by mistake!"

Patient.—" Quick, quick! Let me swallow a piece of blotting paper!"

